# First comprehensive report of bacteria spp. associated with cloaca of *Laudakia nupta* (Sauria: Agamidae) in Iran using molecular studies

**DOI:** 10.1186/s42826-019-0001-5

**Published:** 2019-06-24

**Authors:** Farkhondeh Sayyadi, Nasrullah Rastegar-Pouyani, Mehri Azadbakht, Khosrow Chehri

**Affiliations:** Department of Biology, Faculty of Basic Sciences, Razi University, Taq-e Bostan, Bagh-e Abrasham, Kermanshah, 6714967346 Iran

**Keywords:** *Bacillus*, *Pseudomonas*, Cloaca, Lizard, Bacteria, *Laudakia nupta*

## Abstract

Iran bears a remarkable variety of reptiles. One of the lizard families occurring in Iran is the Family Agamidae which is widely are distributed throughout the old world. The large-scaled rock agamid, *Laudakia nupta*, is one of the well-known agamid. There are few reports of cloacal microbial on reptiles hence their function in cloacae remains unknown. *Laudakia nupta* usually live in rural and urban areas and close vicinity to man, they are likely to play an important role in the spread of disease that may be caused by these microorganisms and their transmission to man. Therefore, the aim of this study was to identify the bacterial flora colonizing the cloacal region of *Laudakia nupta* using molecular studies. The cloacal fluids were directly placed on nutrient agar (NA) plates and incubated at 25 ± 2 °C for 48 h. The resulting bacterial colonies were transferred to fresh nutrient agar (NA) plates for molecular studies. Twelve isolates were obtained from 17 specimens of *Laudakia nupta.* All bacteria isolates were identified as *Bacillus subtillis* (5), *Bacillus cereus* (4), *Bacillus* sp. (1)*, Pseudomonas putida* (1), and *Pseudomonas* sp. (1) based on partial sequences of the *16 s rRNA* gene. This is the first comprehensive report of bacteria spp. associated with cloaca of *Laudakia nupta* using molecular studies. In this research, we found that *Laudakia nupta* can be a carrier of bacteria which can transfer microorganisms to hosts.

## Introduction

Reptiles are cold-blooded vertebrates among the oldest amniotes and are highly diverse in their morphology and ecological niches [1]. The Agamidae, a monophyletic family of lizards, are distributed throughout the old world [2]. In Iran, the family Agamidae encompass at least four genera: *Laudakia*, *Calotes*, *Trapelus* and *Phrynocephalus* [3, 4]. The large- scaled rock lizard is commonly referred to as *Laudakia nupta.* They are abundant on and among large rocks having deep crevices and around human habitation, commonly seen on walls, mud-brick dwellings, and the tombs and monuments of graveyards [3–6]. Reptiles have usually microbial contamination and asymptomatic carriers to transmission of microorganisms [7]. Reptiles, even if appear healthy, often able to carry a wide variety of pathogens that can infect humans [8, 9]. Viral, protozoal, fungal and parasitic agents can infect the cold-blooded animals, but bacteria are the most common pathogens recovered from these animals and in most cases they can be transmitted to humans [9]. Some bacteria are cause of infectious pathologies in reptiles, but often represent the normal bacterial flora of these animals [9]. Also different species of lizards have also been associated various bacteria species for example, there are a number of bacteria in the cloaca of giant lizards, including *Citrobacter* spp., *Enterobacter* spp., *Escherichia coli*, *Klebsiella oxytoca*, *Salmonella* spp., *P. aeruginosa*, *Corynebacterium* spp., *Staphylococcus* spp., and *Streptococcus* spp. [10]. The main bacterial infections, transmitted from reptiles to humans are salmonellosis, mycobacteriosis, chlamydophilosis, Aeromonas and Pseudomonas infections [9]. *Pseudomonas* is Gram negative microorganisms widely spread in the environment and considered opportunistic pathogens for animals and humans [9]. *P.* spp. usually cause infections of gastrointestinal tract in human and animal [11–13]. *Bacillus* is Gram-positive, aerobic, ubiquitous bacteria that live in every natural environment [11–14]. Humans may come in contact with *B.* spp. that may cause illness in them [9]. *B. cereus* can result in intoxications that cause nausea, vomiting, and abdominal cramps or diarrhea [11–13].

*L. nupta* are plentiful in rural and urban areas and live in close vicinity to man, they are likely to play an important role in the spread of disease that may be caused by these bacteria and transmission to man. Therefore, identification the bacterial flora associated with cloaca of *L. nupta* is important. The aims of this study were to characterize bacterial flora cloacal sites in rock lizard agama. This study has helped us to understand the normal bacterial flora in *L. nupta*.

## Results

Our objective was to demonstrate the bacteria present in the cloacal fluid of *Laudakia nupta* that observed under the stereo microscope. Twelve isolates of aerobic bacteria were obtained from cloacae. All isolates were chosen for DNA sequence analysis using the *16 s rRNA* gene. After molecular studies, DNA extraction and PCR reaction, all bacteria isolates were identified as *B. subtillis* (5), *B. cereus* (4), *Bacillus* sp. (1)*, P. putida* (1), and *Pseudomonas* sp. (1). Then obtained sequences were compared with those on the NCBI. From similarities searched at NCBI database, identification of all species was confirmed with statistical significance.

## Discussion

The results of this investigation demonstrate the aerobic bacterial flora in the cloacae of rock lizard agama, *L. nupta*. During recent years some reptiles including turtles, snakes and lizards are kept in domestic environment and often bred in houses [9]. *L. nupta* usually lives in rural and urban areas and in the vicinity of humans [15]. Reptiles although clinically healthy, often carry and transmit opportunistic pathogens such as bacteria which can become cause of serious infections [9]. The bacterial species including *Stenotrophomonas maltophilia*, *Pseudomonas spp*., *Citrobacter spp*., *Enterobacter spp*., *Escherichia coli*, *Proteus spp., Serratia spp*., and *Salmonella spp*. isolated from healthy captive green iguanas that can be a potential health risk for humans [8–19]. Our results showed that *B. subtillis, B. cereus, Bacillus* sp*., P. putida,* and *Pseudomonas* sp*.* occurred in the cloaca of *L. nupta. Pseudomonas* spp. is widespread in the environment and considered opportunistic pathogens for animals and humans [9]. *Pseudomonas* spp*., Citro bacter*spp*., Proteus vulgaris, Enterobacter* spp*., Serratia* spp*.,* and *Salmonella spp.*are capable of causing opportunistic infection in reptiles [8–22]. *Pseudomonas* spp. cause infections of urinary tract, respiratory system, skin, soft tissue, bone, joint and gastrointestinal tract [9–23]. *P. putida,* and *Pseudomonas* sp*.* were present in our study and are thus a potential risk.

*Bacillus* sp*.* has been introduced as the cloacal bacterial flora of the snake *Elaphe quatuorlineata*, [24]. Most of the isolated bacteria such as *Bacillus* sp*.* have already been described as causes of infection in both reptiles and humans. *B.cereus* was also identified as opportunistic pathogen of humans [25]. In our study *B. subtillis B. cereus* and *Bacillus* sp*.,* were part of the natural cloacal flora of the agamid, *L. nupta*. Humans may come in contact with *Pseudomonas* and *Bacillus* bacteria from the environment and directly from spreader animals [9–23]. Therefore, reptiles can serve as the carriers for the transmission of microorganisms such as bacteria to other animals and humans.

In reptiles, the microbial flora may not be pathogenic for their natural hosts but it could be dangerous and pathogenic if they get contact with humans particularly elderly, pregnant women, those with a weaken the immune system, and other animals [9]. In fact, it can be claimed that most infections and contamination of reptiles are due to pathogenic bacteria and since *L. nupta* live in close vicinity to man, they are likely to play an important role in the spread of various diseases. Therefore, identification of the normal bacterial flora associated with cloaca of *L. nupta* is important.

## Conclusions

In conclusion, the results of this study indicate that rock lizard *L. nupta* harbor some bacteria in cloacal region. Many isolated bacteria have already been described as causes of infection. To the author ʼ s knowledge this is the first survey of bacterial flora colonizing the cloacal region of rock lizard. By doing this research, we found that *L. nupta* can be a carrier of microorganisms which can transfer bacteria to the environment, other animals, and humans. This study only introduce and identification of bacteria associated with cloacal region of the rock lizard *L.nupta* and it is recommended that the pathogenesis tests be performed on bacterial flora in this region.

## Materials and methods

This research was approved by the ethics committee (95.01.20) of Razi University for 17 specimens of *L. nupt.*

### Isolation of bacterial species

**Fig. 1:**
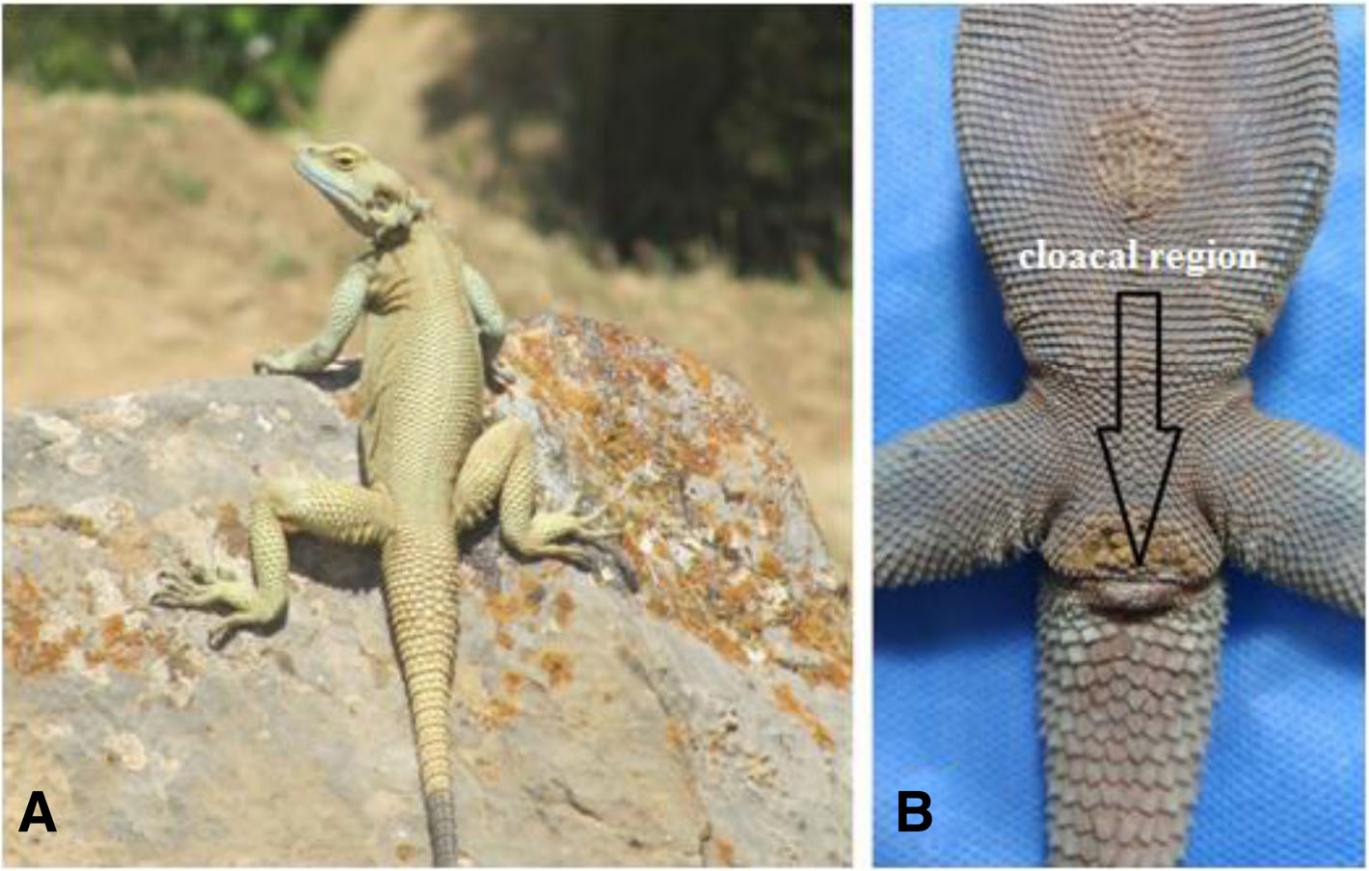
**a** *Laudakia nupta* in natural habitat. **b** Cloacal area in *Laudakia nupta.↓*: Cloacal region

From May to September 2017, 17 specimens of *L.nupta* (male and female) collected from Kermanshah Province, Western Iran (Fig.1a). The lizards were taken from the mountainous areas, among rocks and crevices and ruins. The collected lizards were identified based on key identification. The animals were transferred to the laboratory. Also in the laboratory, sufficient light, optimum temperature, water and food were provided for samples. During the survey of cloacal region of *L. nupta*, we noticed the presence of microorganisms such as bacteria in this region under the stereo microscope (Fig.1b). The exit part of cloacal region of samples was disinfected by ethylic alcohol (70%). The cloacal fluid from inside of cloacal region were directly placed on nutrient agar (NA) plates and incubated at 25 ± 2 °C for 48 h. The resulting bacterial colonies were transferred to fresh nutrient agar (NA) plates for further studies.

### DNA extraction, PCR amplification, and sequencing alignment

The DNA extraction was done by alkaline lysis method [16]. The DNA amplification of the *16 s rRNA* gene was conducted using two universal primers 27F (5-AGAGTTTGGATCMTGGCTCAG-3) and 1429 (5-CGGTTACCTTGTTACGACTT-3) [17]. 50 μl of PCR product was sent to company Bioneer (Korea) for sequencing. The PCR mixture was centrifuged for 30 s and tubes were transferred to the Thermal Cycler Apparatus. The reaction of PCR was prepared in the total volume of 50 μl in the eppendorf tubes. Nucleotide sequences were manually edited and assembled with BioEdit software version 5.0 (bioedit.software.informer.com). The aligned sequences were BLAST in genome database of GenBank to identify all the selected isolates.The edited *16 s rDNA*gene sequences were compared with other available bacterial species sequences in the GenBank [18].

## Abbreviations

*B*: *Bacillus*
*L*.*nupta*: *Laudakia nupta*
*P*: *Pseudomonas*

## Authors’ information

Farkhondeh Sayyadi: Ph.D. student of biology in Razi University of Kermanshah, Iran.

Nasrullah Rastegar-Pouyani: Professor of Zoology in Razi University of Kermanshah, Iran.

Mehri Azadbakht: Assistant Professor of Anatomy in Razi University of Kermanshah, Iran.

Khosrow Chehri: Associate Professor of Pharmacology in Razi University of Kermanshah, Iran.
